# Magnetic Properties of Prolate Lanthanide Complexes: Synergy between Axial and Equatorial Ligands

**DOI:** 10.1021/acs.inorgchem.5c01521

**Published:** 2025-06-24

**Authors:** Leonardo Tacconi, Anna S. Manvell, Arianna Lanza, Høgni Weihe, Maher Hojorat, François Riobé, Olivier Maury, Jesper Bendix, Mauro Perfetti

**Affiliations:** † Department of Chemistry “Ugo Schiff”, DICUS and INSTM Research Unit, 9300 University of Florence, Sesto Fiorentino, Florence, 50019, Italy; ‡ Department of Chemistry, 4321 University of Copenhagen, Universitetsparken 5, Copenhagen DK-2100, Denmark; § CNRS, ENS de Lyon, LCH, UMR 5182, 69342, Lyon cedex 07, France; ∥ Univ. Bordeaux, CNRS, Bordeaux INP, 129863 ICMCB, UMR 5026, Pessac F-33600, France

## Abstract

The chemical design of single-molecule magnets is a vibrant field of research. Huge efforts in determining the key factors to slow down the relaxation time of the magnetization led to massive improvements in the blocking temperature. Even though several lanthanides are excellent magnetic anisotropy building blocks, the current literature is dominated by (pseudo)-axial Dy^3+^ complexes. Prolate ions such as Er^3+^ and Yb^3+^ exhibit on average relatively modest barriers and fast relaxation times. In this paper, we pinpoint how the combination of symmetry and ligand charge distribution affects the slow relaxation of prolate ions. In this paper, we synthesized and characterized two new lanthanide complexes, namely, [LnDOTA]­[CoF_2_(py)_4_] (Ln = Er^3+^ or Yb^3+^, DOTA = tetraazacyclododecane-*N*,*N*′,*N*″,*N*‴-tetraacetate, py = pyridine). Such complexes were used to provide a proof of concept that apical ligands, typically considered detrimental in prolate ion complexes, can be exploited in combination with the other ligands’ geometry to improve the performances of the system.

## Graphical abstract

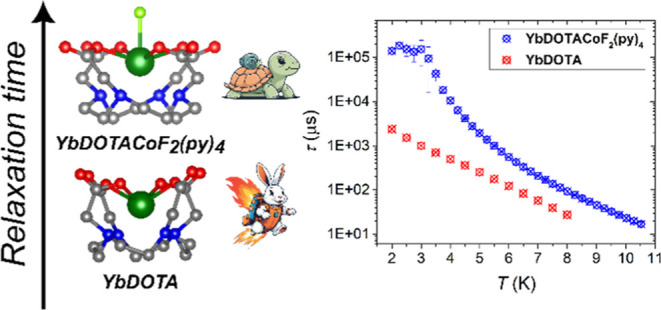

## 1 Introduction

Designing Single Molecule Magnets (SMM) is a growing research field.^1,2^ One of the most important properties to design is magnetic anisotropy, *i.e.*, the different magnetic response of a material depending on the spatial direction, dictated by a combination of metal ion and ligand field potential.^3^ Lanthanide ions are excellent anisotropy building blocks due to the inner nature of the partially filled 4f orbitals preventing the quenching of the orbit.^4^ A few years ago, simple design principles were extracted: to stabilize the maximum projection of the total angular momentum (*J*), ions exhibiting an oblate electron density (Ce^3+^, Pr^3+^, Nd^3+^, Tb^3+^, Dy^3+^, Ho^3+^) should be coordinated with ligands bearing negative charges in axial positions, while ions exhibiting prolate electron density (Pm^3+^, Sm^3+^, Eu^3+^, Er^3+^, Tm^3+^, Yb^3+^) should optimally have a strong equatorial ligand field.^5,6^ Even though the model does not account for other potentially important contributions such as covalency, it was used with great success by many researchers, prompting the realization of huge barriers for the relaxation of the magnetization.^7–9^ However, only a few SMM possess dynamics slow enough to be detected at high temperature, due to undesirable relaxation processes within the low-lying states, such as Quantum Tunnelling of the Magnetization (QTM) and spin-phonon relaxation.^10–12^ The preferred strategy to reduce the QTM is to increase the symmetry of the ligand field and to reduce the number of CF parameters able to mix the states and enhance the states purity, generating states less prone to be mixed by the fluctuating magnetic fields produced by the neighboring molecules.^13–15^ While remarkable results have been achieved with Dy-based molecules,^7,8,16^ their use for engineering SMM based on prolate ions such as Er^3+^ and Yb^3+^ has been less satisfactory.^17^

In this paper, we present and investigate two Er- and Yb-based complexes that have been designed to be tetragonal and to bear charges in both axial and equatorial positions. The molecules that we present here can be described with the general formula [LnDOTA]­[CoF_2_(py)_4_] (Ln = Er^3+^ or Yb^3+^, DOTA = tetraazacyclododecane-*N*,*N*′,*N*″,*N*‴-tetraacetate, py = pyridine, **ErCo** and **YbCo** hereafter). The structures of the complexes are reported in Figure 1. The 4-fold symmetry of the apical, charge-compensating ligand installs an overall tetragonal symmetry to the binuclear complex, while the charge distribution surrounding the lanthanide ion is expected to be dominated by the equatorial oxygen atoms, as previously calculated for the DOTA ligand.^18^ This ligand field is usually considered suboptimal for oblate ions. Accordingly, the Dy derivative was reported to exhibit an easy plane-like magnetic anisotropy, and no slow relaxation of the magnetization was detected.^19^ By comparing the results obtained on our molecules with those obtained on previously reported similar complexes, we shed light on how apical ligands can be used on prolate ions to increase the relaxation time at a low temperature.

**Figure 1:**
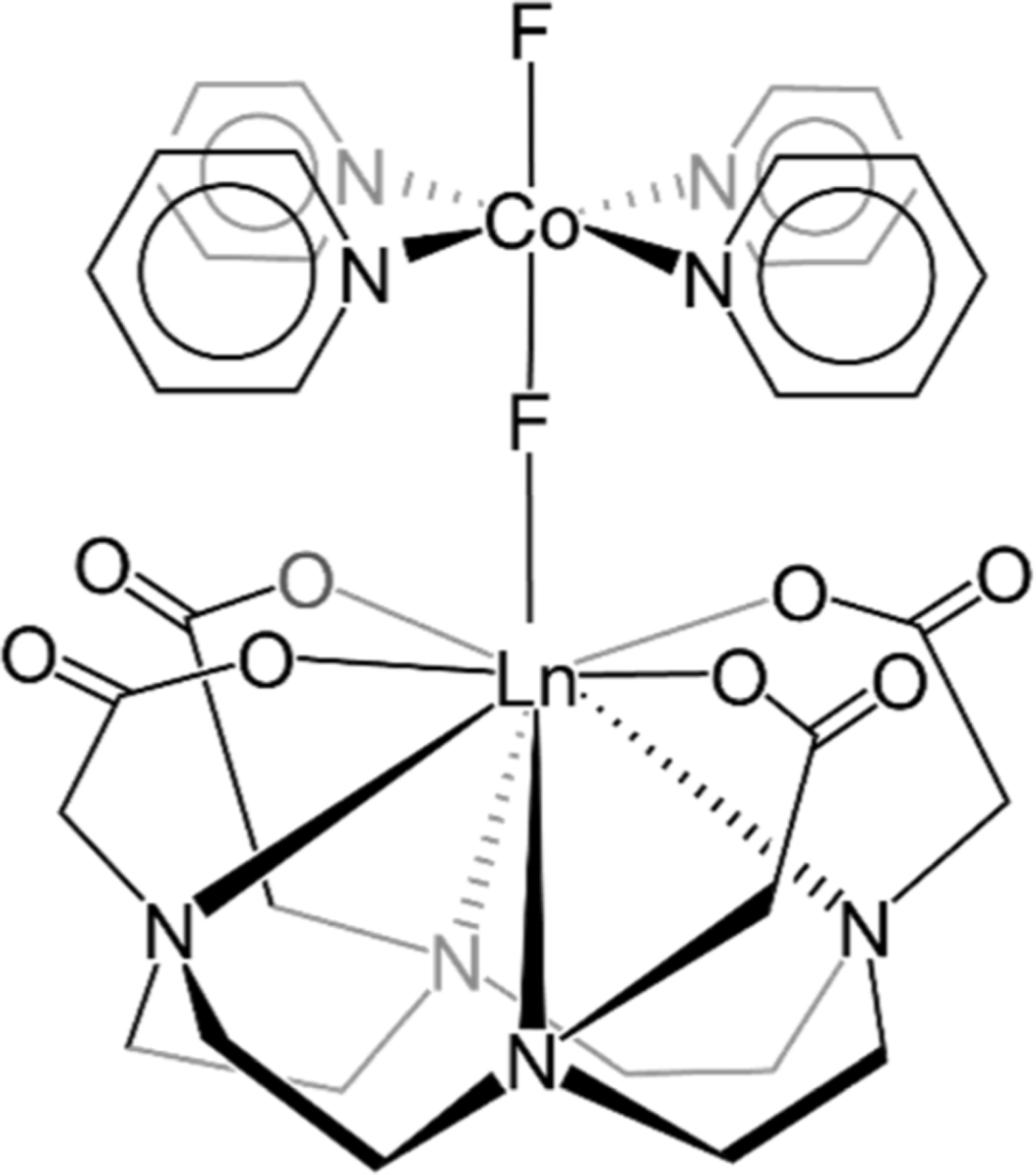
Structure of the **LnCo** complexes (Ln = Er, Yb). The vertical axis is a C_4_ axis of the structure.

## 2 Experimental Section

No uncommon hazards are noted.

### 2.1 Synthesis of the Complexes

The trivalent transition metal building blocks *trans*-[CoF_2_(py)_4_]­(ClO_4_)^20^ and *trans*-[CrF_2_(py)_4_]­(NO_3_)^21^ were synthesized as described in the literature. Metathesis of the cobalt perchlorate to nitrate is preferable for the following syntheses. This was achieved by metathesis from a slightly acidic aqueous solution with a slight excess of (C_4_H_9_)_4_N­(NO_3_) (Sigma-Aldrich), followed by filtering and slow evaporation to near dryness. CAUTION: perchlorate salts are potentially dangerous and must be handled with care.

#### 2.1.1 Synthesis of [Er­(DOTA)]­[CoF_2_(py)_4_]·10H_2_O (ErCo)

H_4_DOTA (100 mg, 0.25 mmol) and ErCl_3_·6H_2_O (96 mg, 0.25 mmol) were dissolved in 2.5 mL of water. The solution was then heated to 80 °C, and 0.75 mL of a solution of NaOH (40 mg, 1.0 mmol) was added dropwise. If the resulting solution showed remnant turbidity, a few extra milligrams of H_4_DOTA were added. The resulting solution was then filtered, and the volume was reduced to ca. 2 mL and left to cool. Then, a saturated solution of *trans*-[CoF_2_(py)_4_]­(NO_3_) (118 mg, 0.25 mmol) was added. The resulting solution was filtered and left for crystallization. After around 12 h, square, pink crystals were formed, suitable for X-ray diffraction. The yields in the procedure are only around 30%. Decreasing the volume to 60% of the solution indicated above and allowing 48 h for crystallization increase the yield to ca. 50% (140 mg). Elemental analysis: calculated for C_36_H_64_CoF_2_N_8_O_18_Er: C: 37.24%, H: 5.56%, N: 9.65%; found: C: 34.96%; H: 5.66%, N: 9.64%.

#### 2.1.2 Synthesis of [Yb­(DOTA)]­[CoF_2_(py)_4_]·11H_2_O (YbCo)

H_4_DOTA (100 mg, 0.25 mmol) and YbCl_3_·6H_2_O (93 mg, 0.24 mmol) were dissolved in 2 mL of water. The solution was then heated to 80 °C, and 0.75 mL of a solution of NaOH (40 mg, 1.0 mmol) was added dropwise. If the resulting solution showed remnant turbidity, a few extra milligrams of H_4_DOTA were added. The resulting solution was then filtered, and the volume was reduced to ca. 1.5 mL and left to cool. Then, a saturated solution of *trans*-[CoF_2_(py)_4_]­(NO_3_) (118 mg, 0.25 mmol) was added. The resulting solution was filtered and left for crystallization. After around 12 h, square, pink crystals were formed, suitable for X-ray diffraction. The yields in the procedure are around 45%. Elemental analysis: calculated for C_36_H_68_CoF_2_N_8_O_19_Yb: C: 36.43%, H: 5.77%, N: 9.44%; found: C: 36.13%; H: 5.63%, N: 9.88%.

#### 2.1.3 Synthesis of [Yb­(DOTA)]­[CrF_2_(py)_4_]·11H_2_O (YbCr)

H_4_DOTA (205 mg, 0.51 mmol) and YbCl_3_·6H_2_O (195 mg, 0.50 mmol) were dissolved in 4 mL of water. The solution was then heated to 80 °C, and 1.5 mL of a solution of NaOH (80 mg, 2.0 mmol) was added dropwise. If the resulting solution showed remnant turbidity, a few extra milligrams of H_4_DOTA were added. The resulting solution was then filtered; the volume was reduced to ca. 3.5 mL and left to cool. Then, a saturated, filtered solution of *trans*-[CrF_2_(py)_4_]­(NO_3_) (248 mg, 0.53 mmol) was added. The resulting pink solution was filtered and left for crystallization. After around 12 h, tabular, pink–violet crystals were formed, suitable for X-ray diffraction. The yields in the procedure are only around 35%. Decreasing the volume to two-thirds of the solution indicated above and allowing 48 h for completion of the crystallization increase the yield to 400 mg (68%). Elemental analysis: calculated for C_36_H_68_CrF_2_N_8_O_19_Yb: C: 36.64%, H: 5.81%, N: 9.50%; found: C: 34.33%; H: 5.80%, N: 9.54%.

### 2.2 Structural Characterization

Data for single-crystal X-ray diffraction were collected on a Bruker D8 VENTURE diffractometer equipped with a Mo Kα X-ray source (λ = 0.71073 Å) and a PHOTON 100 CMOS detector. For temperature control, the diffractometer is equipped with an Oxford Cryosystem Cryostream 800. All measurements were done at 100 K. Crystals were mounted with a small amount of silicone grease on kapton loops. Data reductions were performed in the APEX4/5 software, and the absorption correction was done with the multiscan method SADABS. Structure solutions and refinement were done in the Olex2 software with the ShelXT package included.^22,23^ Powder X-ray diffraction data were collected on a Bruker D8 ADVANCE powder diffractometer equipped with a Cu Kα X-ray source (λ = 1.5418 Å).

### 2.3 Magnetic Characterization

The samples were investigated as polycrystalline samples, and to circumvent field-induced orientation of the crystallites, they were analyzed as pressed pellets. Temperature- and field-dependent direct current (DC) magnetic measurements were conducted using a Quantum Design MPMS SQUID magnetometer. The raw data were processed in order to remove the contribution of the sample holder and corrected for sample diamagnetism using Pascal’s constants. Alternating current (AC) magnetic susceptibility characterization was conducted using Quantum Design PPMS and Quantum Design SQUID magnetometers, in the ranges of 10 Hz–10 kHz and 10 Hz–1.5 kHz, respectively. The AC data were subsequently analyzed and fitted with an extended Debye model using a previously described code.^24^

### 2.4 Fitting Procedure of Crystal Field Parameters

Crystal field parameters for **ErCo** and **YbCo** were obtained through a fitting procedure of magnetic and luminescence data (just for **YbCo**) using a custom MATLAB code based on the EASYSPIN package,^25^ to simulate the magnetic properties, and the fminuit minimization routine,^26^ to minimize an object function *f*(*x*). The function to be minimized was the error between the experimental and simulated data, evaluated as the root-mean-square relative error (RMSRE).^27^

$$f\left(\overset{\to }{x}\right)=RMSRE=\sqrt{\frac{1}{n}\overset{n}{\underset{i=1}{\sum }}{\left(\frac{x_{i}-y_{i}}{y_{i}}\right)}^{2}}$$

*x*_*i*_ and *y*_*i*_ represent the simulated and experimental values, respectively, while *n* is the number of points of the data set.

The initial guess of the fitting procedures was taken from the crystal field parameters reported for the isostructural Dy^3+^ complex.^19^ In particular, the transferability of CF parameters in Wybourne’s notation between different tripositive Ln ions was accounted for. Then, parameters in Wybourne’s notation were converted to Steven’s using the tabulated α­(*J*), β­(*J*), and γ­(*J*) constants.^28^

### 2.5 Luminescence Spectroscopy

The luminescence spectra were measured using Horiba–Jobin–Yvon Fluorolog-3 fluorometers. The crystalline powders in EPR quartz tubes were excited by unpolarized light from a 450 W xenon continuous wave lamp and detected at right angles through a FGL850 high-pass filter by using a liquid nitrogen cooled Symphony II CCD-camera iHR320 series (emission spectra). Spectra were corrected for both excitation source light–intensity variation (lamp and grating) and emission spectral responses (detector and grating). The spectra at 77 K were measured by submerging the sealed tubes in a liquid-nitrogen-filled quartz dewar flask.

## 3 Results and Discussion

### 3.1 Structural Characterization

Single-crystal X-ray diffraction (SC-XRD) analysis demonstrated that both **YbCo** and **YbCr** crystallize in the tetragonal space group *P*4/*n* (n. 85). This finding is analogous to that of the previously reported isostructural **YCr**- and **DyCr**-based complexes. However, the diffraction data acquired on the **ErCo** complex resulted in a different space group, specifically *P*4/*ncc* (n. 130), which is also similar to the observations made for the **DyCo**-based complex.^19^ In both cases, the metal complex is located on a proper C_4_ axis, with the symmetry axis passing through the lanthanide-transition metal direction. A comparison between the crystal packing in the two space groups is reported in Figure S1 and Table S1, while powder X-ray diffractograms acquired on the three derivatives are shown in Figures S2–S4.

### 3.2 Magnetometric Characterization

The magnetic properties of the two complexes **ErCo** and **YbCo** are dictated by the tripositive lanthanide ion because Co^3+^ in an octahedral environment usually is a 3d^6^ low spin diamagnetic ion. To gain insights into the electronic structure and magnetic behavior of the lanthanide ions, microcrystalline powder samples were investigated using SQUID magnetometry. The temperature dependence of the product between the magnetic susceptibility and the temperature (χ*T*) is presented in Figure 2a. At room temperature, the value of χ*T* reaches 11.54 and 2.22 emu K mol^–1^ for **ErCo** and **YbCo**, respectively. The value for **ErCo** is in accordance with the predictions of the Curie law (*J* = 15/2, *g*_*J*_ = 6/5, χ*T* ^Curie^ = 11.58 emu K mol^–1^), while **YbCo** exhibits a more pronounced deviation (*J* = 7/2, *g*_*J*_ = 8/7, χ*T* ^Curie^ = 2.57 emu K mol^–1^), indicating that the total splitting of the *J* = 7/2 ground state is sufficiently large to avoid reaching the Curie limit. As the temperature is reduced, a monotonic decrease of the χ*T* value is observed for both derivatives, a common feature associated with the progressive depopulation of the *m*_*J*_ levels.^29^

**Figure 2:**
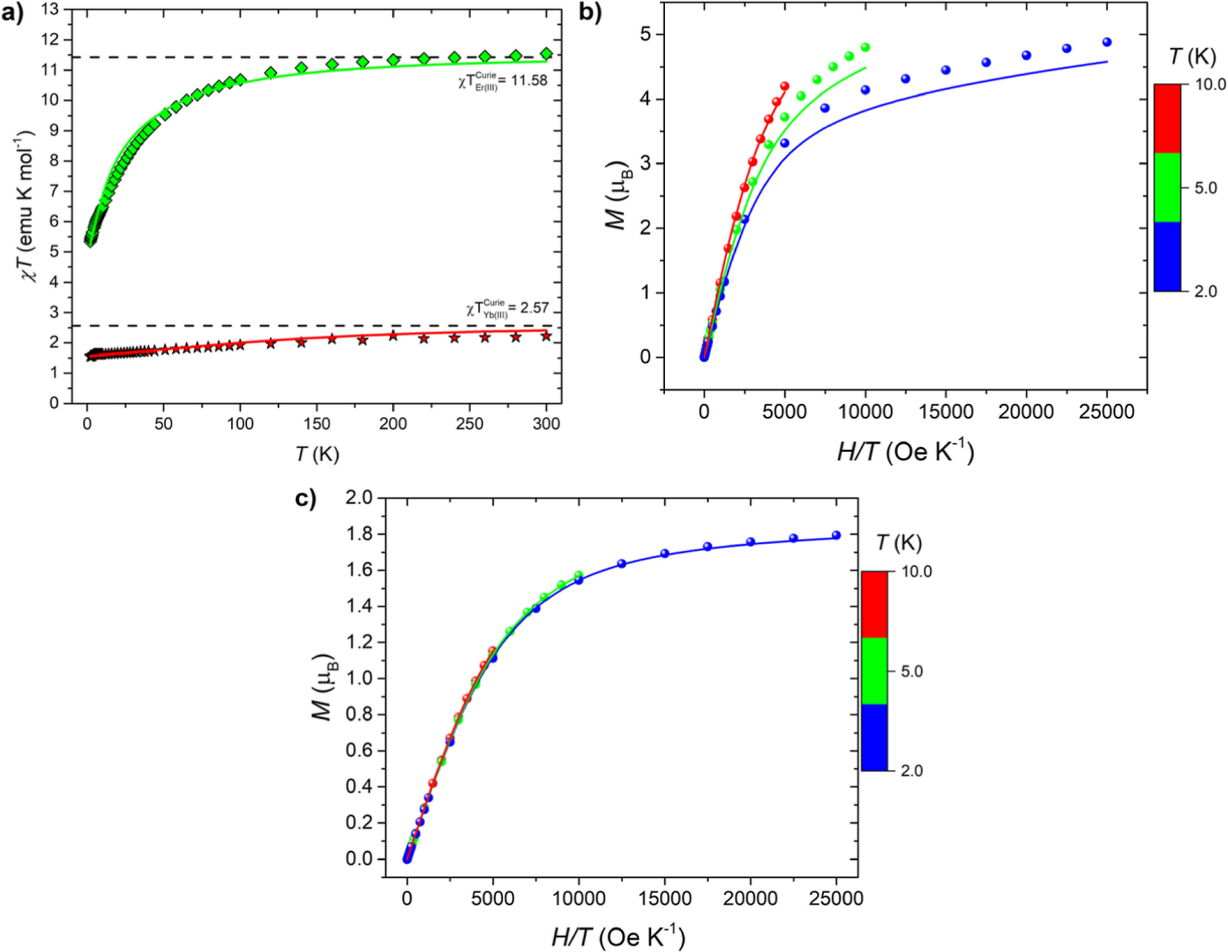
(a)­Temperature dependence of the χ*T* for **ErCo** (green) and **YbCo** (red) acquired at *H* = 1000 Oe. Solid lines are the best fit (see text), while black dashed lines represent the Curie constant for the lanthanide ions. Low-temperature reduced magnetization curves acquired on **ErCo** (b) and **YbCo** (c). Solid lines are the best fit (see text).

The reduced magnetization curves for **ErCo** do not superimpose (Figure 2b), indicating the presence of low-lying energy levels. In contrast, the superimposable curves observed for **YbCo** (Figure 2c) suggest that the ground state is well separated from the excited states. Notably, **ErCo** also shows a hysteretic behavior at 2 K (see Figure S5).

### 3.3 Luminescence Spectroscopy

To gain a more detailed understanding of the energy structure of **YbCo** and the reasons behind its room temperature χ*T* being so different from the Curie value, low-temperature (77 K) luminescence spectroscopy was performed on a polycrystalline sample. Unfortunately, **YbCo** exhibited no luminescence, and no signal was detected for the ^2^ *F*_5/2_→^2^ *F*_7/2_ transition. Accordingly, the same experiment was conducted for the isostructural **YbCr** derivative. Importantly, the **DyCr** derivative, already reported by some of us, showed an electronic structure similar to its **DyCo** analogue.^19^ In this instance, a discernible signal was observable within the near-infrared (NIR) range (Figure S6). Although there are more peaks than expected from the *J* = 7/2 ground multiplet of Yb^3+^, a feature already reported for other Yb^3+^-based complexes,^30–32^ four main transitions were identified, providing an estimate of the crystal field splitting of the ground state. The total crystal field splitting was determined to be ≈540 cm^–1^.

### 3.4 Single Molecule Magnet Behavior

The magnetization dynamics of the three samples was investigated by using temperature- and magnetic-field-dependent AC magnetometry. The frequency dependence of the real (χ^′^) and imaginary (χ^″^) components of the magnetic susceptibility was fitted with an extended Debye model^33^ (see Experimental Section for additional information). Single or double-component Debye models were employed depending on the number of detectable relaxation pathways.

None of the samples exhibited slow relaxation dynamics in the absence of an external magnetic field. This is very common for Er^3+^ complexes,^34,35^ with a few exceptions based mainly on the cyclooctatetraenyl^36,37^ (COT^–^) or low coordinated architectures,^38,39^ and to the best of our knowledge, only one Yb^3+^ complex has been recorded to exhibit slow relaxation of the magnetization without an applied static field.^40^ However, the application of a modest magnetic field at 2 K is sufficient to slow the relaxation in both **ErCo** and **YbCo**.

At low fields, **ErCo** (Figure S7) exhibits a distinct peak in χ^″^(ν) curves, indicative of a single relaxation pathway (Relaxation Pathway 1, RP1). As the magnetic field is increased to 3000 Oe, another process emerges at low frequency (Relaxation Pathway 2, RP2). A further increase in the field results in RP1 becoming faster, suggesting dominance of a direct process for RP1. Conversely, an increase in the magnetic field resulted in a slowing of RP2 (Figure 3a), which can be attributed to a suppression of the QTM dynamics. The temperature dependence of the relaxation dynamics of **ErCo** was investigated at 1000 Oe (Figure S8). RP1 is already beyond the experimental frequency range at 2 K; thus, its thermal evolution cannot be traced. Conversely, upon increasing the temperature, the low-frequency RP2 is accelerated (Figure 3b), suggesting the involvement of thermally active relaxation mechanisms.

**Figure 3:**
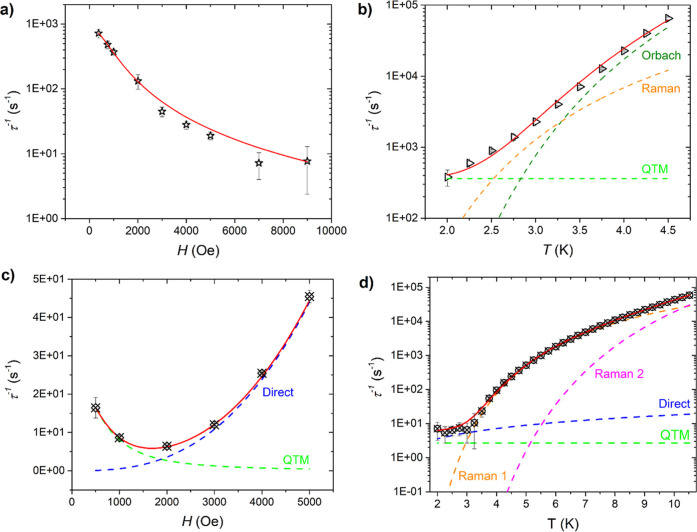
Magnetic field and temperature dependences of the experimental (black symbols) relaxation frequency τ^–1^ of **ErCo** (a,b) and **YbCo** (c,d) fitted with the models discussed in the main text. Dashed lines represent the single contributes to the relaxation, while solid red lines are the sum of all processes.

The magnetic field and temperature dependences of RP2 were then fitted with the following model.

$$\tau^{-1}\left(H,T\right)=\frac{B_{1}}{1+B_{2}\cdot H^{2}}+R_{1}\frac{e^{h\nu_{1}/k_{B}T}}{{\left(e^{h\nu_{1}/k_{B}T}-1\right)}^{2}}+\frac{1}{\tau_{0}}e^{\left(-\Delta /k_{B}T\right)}$$ 1

The three terms represent the QTM, Raman, and Orbach processes, respectively.^41^ A direct process was not included in the model given the observation of a monotonous decrease in τ^–1^(*H*). To avoid overparametrization during the fitting process, the fitting was conducted in a step-by-step manner. First, the magnetic dependence at 2 K was fitted considering only the QTM process; *i.e*., no thermal process is active in these conditions. Then, the thermal dependence was fitted to eq 1, with the parameters of the QTM process fixed to those previously determined. It is important to note that the parameter Δ was also kept fixed to the energy separation between the ground and first excited states previously determined by the Hamiltonian modeling (see after). The results of the fitting are shown in Figure 3c, while best-fit parameters are reported in Table S2.

The application of an external magnetic field has been observed to suppress the QTM process also for **YbCo**, resulting in a discernible peak in the χ^″^(ν) curves (Figure S9). An increase in the strength of the magnetic field results in an initial shift of the peak to lower frequencies, while the peak moves to higher frequencies when the magnetic field strength exceeds 2000 Oe. This can be attributed to both a suppression of QTM and an increase in the prevalence of a direct process. The thermal dependence of the relaxation dynamics was investigated in an applied magnetic field of 2000 Oe (Figure S10). As the temperature is increased, the peak is observed to move to higher frequencies, indicative of the presence of thermally driven processes. This is consistent with the thermal dependence of the α parameter of the extended Debye model (Figure S11), which shows an initial value coherent with a QTM process and a decrease in temperature, suggestive of the emergence of thermally driven processes.

Therefore, both the magnetic field and temperature dependences of the relaxation frequency τ^–1^ of **YbCo** were modeled employing the following model, previously used on the YbDOTA derivative.^42^

$$\tau^{-1}\left(H,T\right)=\frac{B_{1}}{1+B_{2}\cdot H^{2}}+DH^{m}T+R_{1}\frac{e^{h\nu_{1}/k_{B}T}}{{\left(e^{h\nu_{1}/k_{B}T}-1\right)}^{2}}+R_{2}\frac{e^{h\nu_{2}/k_{B}T}}{{\left(e^{h\nu_{2}/k_{B}T}-1\right)}^{2}}$$ 2

The four terms of the equation refer to QTM, direct process, and two Raman processes, respectively. The inclusion of an Orbach mechanism was excluded in this case due to the very high energy at which the first excited state lies; see Results and Discussion. The fitting procedure was carried out in a manner analogous to that employed for **ErCo**. The best-fit parameters are presented in Table S3, while the results of the fitting procedure are illustrated in Figure 3d.

In Figure 4, we report a plot of the relaxation time versus temperature of **LnCo** and of the parent complexes not bearing an apical ligand, **LnDOTA** (Ln = Er, Yb). Both sets of complexes are tetragonal by design, but the relaxation time is quite different. At low temperature, the complexes reported in this work exhibit a slow relaxation that is 1 to 2 orders of magnitude slower compared to their counterparts. However, when the temperature is increased, this difference is drastically reduced (in the case of the Er-based complexes, the trend is even reversed). Our experiments evidence that the inclusion of an apical ligand in this ligand environment efficiently increases the relaxation time at low temperature, while at higher temperature, its positive effect is lost (for **ErCo**) or irrelevant (for **YbCo**). We have therefore analyzed the reason behind this behavior.

**Figure 4:**
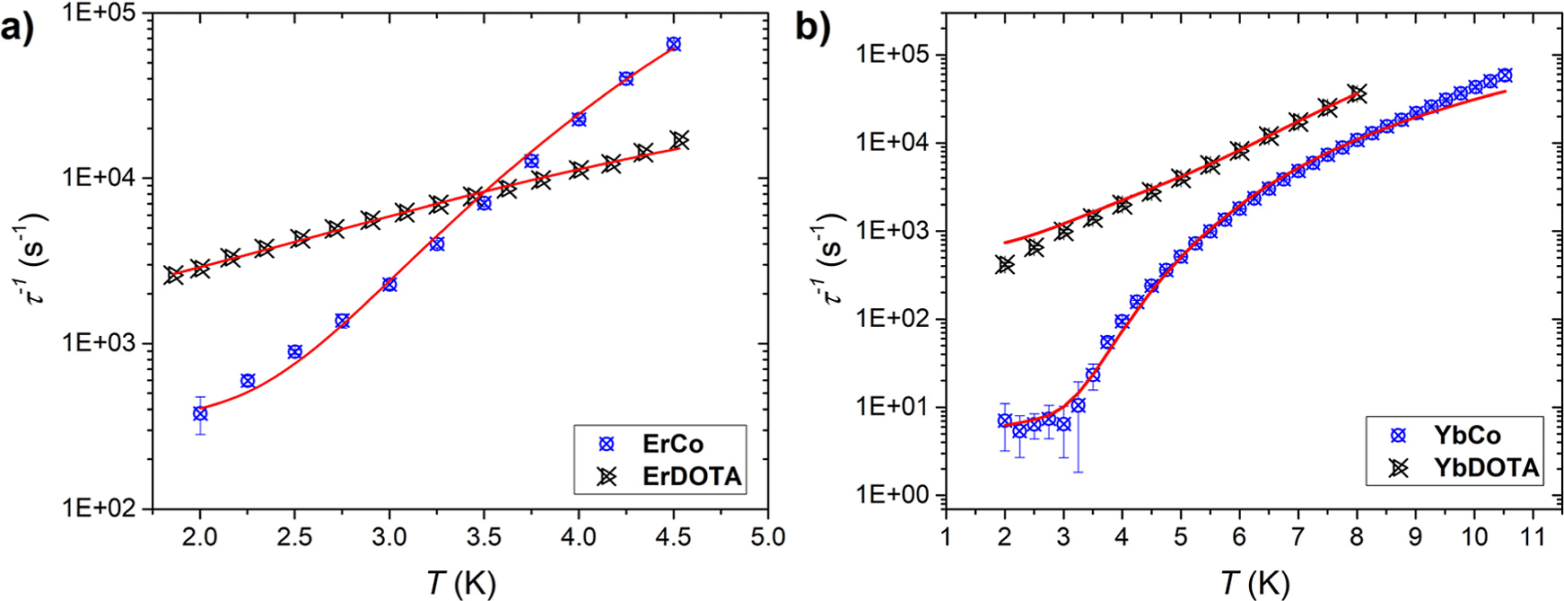
Comparison of the temperature dependence of relaxation frequencies of different LnDOTA complexes: (a) Er-based complexes; (b) Yb-based complexes. Red lines are the best-fit curves.

To gain insight into the electronic structure of the complexes, the experimental data were fitted to extract the Hamiltonian parameters. All the magnetic properties were modeled using the following Hamiltonian.

$${Ĥ}_{\mathrm{Ln}}=\overset{2,4,6}{\underset{k}{\sum }}\overset{k}{\underset{q=-k}{\sum }}B_{k}^{q}{Ô}_{k}^{q}\left(Ĵ\right)+g_{J}\mu_{B}Ĵ\cdot \mathrm{B⃗}$$ 3

The first term refers to the Crystal Field (CF) interaction between the ligands and the metal ion, while the second represents the Zeeman interaction with applied magnetic field *B⃗*. The CF interaction is described by the extended Stevens operators ${Ô}_{k}^{q}$ and a total of 27 B_*k*_^*q*^ parameters. The point group tetragonal symmetry of the complexes allows the number of significant parameters to be reduced to five, including three diagonal (*B_2_^0^*, *B_4_^0^* and *B_6_^0^*) and two out-of-diagonal (*B_4_^4^* and *B_6_^4^*) parameters.^43^ Refined sets of parameters for the three complexes were obtained through a fitting procedure of the DC magnetometry data (see Experimental Section for additional information) and are shown in Table S4. For the **YbCo** derivative, the luminescence data were considered during the fit, *i.e*., the total splitting was imposed to be close to the one recorded experimentally.

In Figure 5, we report a comparison between the electronic structures of **LnCo** and **LnDOTA** (Ln = Er, Yb). As evidenced in Figure 5a,b, the incorporation of an apical ligand results in a modification of the predominant composition of the states. It is noteworthy that tetragonal mixing persists, and the complete composition is shown in Tables S5 and S6. Specifically, the ground state of both **LnCo** derivatives is predominantly composed of the highest *m*_*J*_ level (*i.e*., *m*_*J*_ = 15/2 and 7/2 for **ErCo** and **YbCo**, respectively), while this is not the case for **LnDOTA**. Indeed, the ground-state perpendicular *g* component, which promotes the QTM, is smaller in **LnCo** compared to **LnDOTA** (2.90 vs 3.51 for Er and 0.50 vs 2.90 for Yb). This observation suggests that adding an apical ligand in these systems is effective in reducing the QTM. This phenomenon can be quantitatively evaluated by calculating the matrix element of transition magnetic moment ((|μ_*x*_|+|μ_*y*_|+|μ_*z*_|)/3),^44,45^ reported in Figure 5 and Tables S7–S10. The transition probability associated with QTM is extremely small for both **ErCo** and **YbCo**, while it is significant for **ErDOTA** and **YbDOTA**. Specifically, the probability is notably high in 8-coordinate **YbDOTA**. This behavior may appear counterintuitive; however, the underlying reason can be attributed to the geometrical rearrangement of the complex upon coordination of the apical ligand. The presence of the apical ligand has been shown to exert a pulling force on the lanthanide ion,^42^ displacing it from the DOTA cavity and consequently directing the four charged carboxylic oxygen atoms toward the equator. Concurrently, the donating capacity of the axial ligand is minimal, as reported for the **DyCo** complex, and it does not substantially contribute to the electrostatic potential.^19^ A simple descriptor for such geometrical alterations is the C_4_ axis-Ln-O angle that increases from 62.40° and 61.88° in **ErDOTA** and **YbDOTA**, respectively, to 72.60° and 72.46° in **ErCo** and **YbCo**, respectively. The increased equatorial character of the ligand field is pivotal in stabilizing the high *m*_J_ states. However, it is important to note that the relaxation time of all the studied complexes is modest; therefore, the experimental range of temperatures at which they can be measured with AC magnetometry is limited. Figure 3 evidences that, above 3–4 K, the most important process active in all samples is a Raman relaxation, while the Orbach process can be visible as a tail only in the relaxation of **ErCo**. Furthermore, our model illustrates that the phonon frequencies associated with Raman relaxations are increased in **LnCo** compared to those in **LnDOTA**. Fixing the pre-exponential factors of the Raman processes in **LnCo** to those previously determined for **LnDOTA** leads to the same conclusion (see Tables S11 and S12 for a detailed comparison). This is, in principle, beneficial for the design of a high-temperature SMM, but it is still not sufficient to avoid the dominance of the Raman process over the Orbach one.

**Figure 5:**
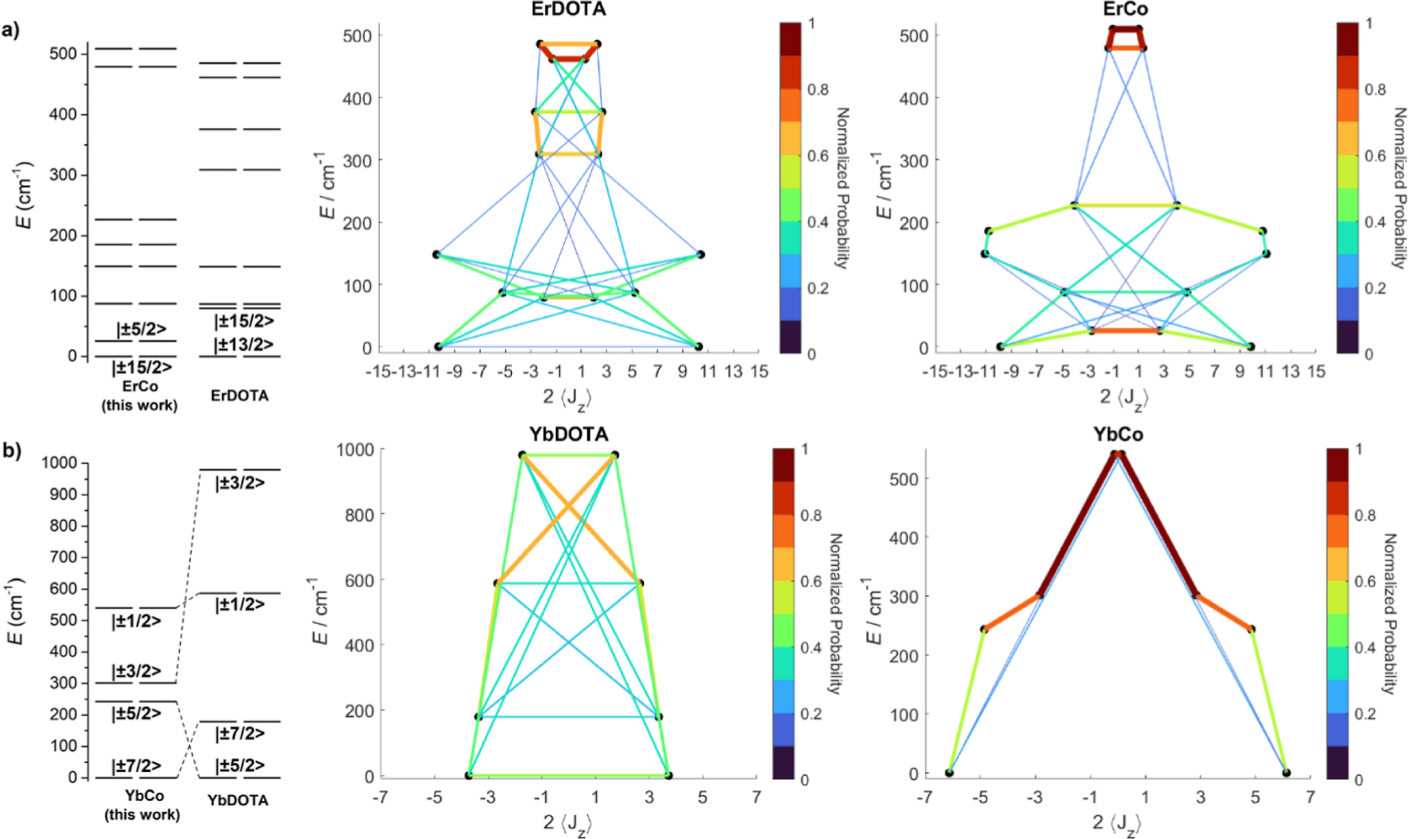
Comparison between the energy structure of **LnCo** and **LnDOTA** based on Er (**a**) and Yb (**b**). Energy structures were simulated using the Hamiltonian parameters obtained from the fitting procedure discussed in the main text for **LnCo** and crystal field parameters reported in literature for **LnDOTA**. Eigenstate energies and *J*_*z*_ expectation values of **ErDOTA**, **ErCo**, **YbDOTA**, and **YbCo** resulting from the discussed Hamiltonian in an applied magnetic field of 1000 Oe. The thickness and color of the lines are proportional to the magnetic transition moments between the states connected by those lines. To ease direct comparison, the transition moments of **ErCo** and **ErDOTA** have been normalized to the highest value calculated for **ErCo**, while transition moments of **YbCo** and **YbDOTA** have been normalized to the highest value calculated for **YbCo**.

## 4 Conclusions

In this paper, we have studied two tetragonal complexes of the DOTA ligand with two prolate ions, Er^3+^ and Yb^3+^. Both complexes showed in-field SMM behavior. The weakly donating apical ligand reduces the QTM by 2 orders of magnitude, demonstrating that our synthetic approach is effective in increasing the performances at low temperature. The phonon frequencies associated with the Raman relaxation are successfully increased in comparison to the complexes devoid of the axial ligand. However, this increase is insufficient to significantly slow down the relaxation time, which remains modest at high temperature. Consequently, we propose that combining the symmetry and steric demands of weakly interacting axial ligands can serve as a tool for tuning and optimization of magnetic relaxation in prolate lanthanide SMMs.

## Supplementary Material



## Notes

Crystal packing of **ErCo** and **YbCo**; PXRD patterns for all samples; hysteresis measurements for **ErCo**; low-temperature luminescence spectrum for **YbCr**; temperature and magnetic field dependence of AC susceptibility curves for **ErCo** and **YbCo**; crystal information for all samples; crystal field parameters for **ErCo** and **YbCo**; detailed energy structure of **ErCo**, **ErDOTA**, **YbCo**, and **YbDOTA**; transition probabilities of **ErCo**, **ErDOTA**, **YbCo**, and **YbDOTA**; and fitting results of AC curves of **ErCo** and **YbCo** (PDF)
