# Supplementary material for: Magnetic Properties of Prolate Lanthanide Complexes: Synergy between Axial and Equatorial Ligands
Source: Inorg Chem. 2025 Jun 24;64(33):16781–8. doi: 10.1021/acs.inorgchem.5c01521 (PMC12381861; doi:10.1021/acs.inorgchem.5c01521)
Supplement: Supplementary file 1 [file ic5c01521_si_001.pdf]

SUPPORTING INFORMATION

The magnetic properties of prolate lanthanide complexes: synergy between axial and equatorial ligands.

Leonardo Tacconi[a], Anna S. Manvell[b], Arianna Lanza[b], Høgni Weihe[b], Maher Hojorat[c], François Riobé[d], Olivier Maury[c], Jesper Bendix[b]*, Mauro Perfetti[a]*

[a]*Department of Chemistry "Ugo Schiff", DICUS and INSTM Research Unit, University of Florence, 50019 Sesto Fiorentino, Florence, Italy*

[b]*Department of Chemistry, University of Copenhagen, Universitetsparken 5, DK-2100 Copenhagen, Denmark.*

[c] *CNRS, ENS de Lyon, LCH, UMR 5182, 69342, Lyon cedex 07, Franced*

[d]*Univ. Bordeaux, CNRS, Bordeaux INP, ICMCB, UMR 5026, F-33600 Pessac, France*

**mauro.perfetti@unifi.it, bendix@chem.ku.dk*

Supporting Figures

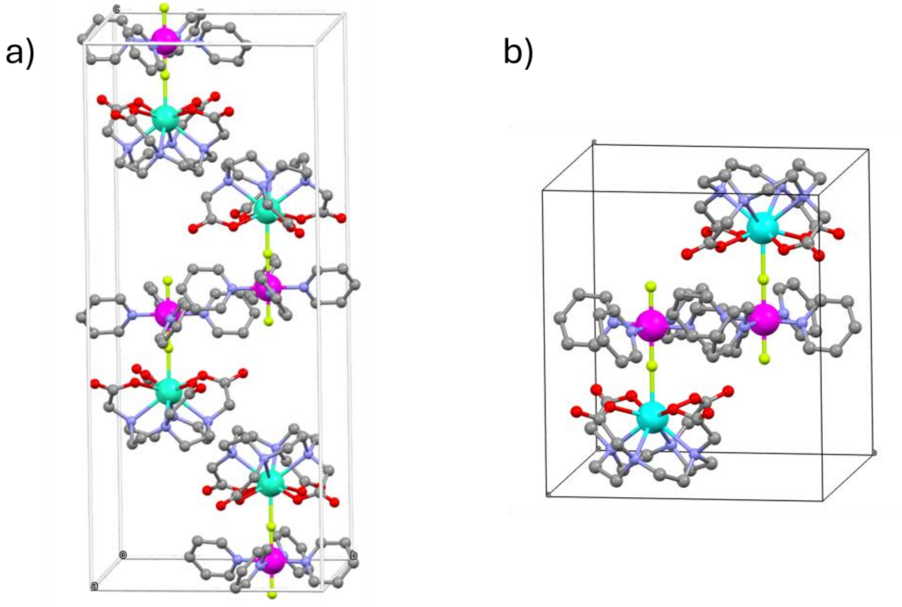

Figure S1 – Crystal packing in the **ErCo** (a) and **YbCo** (b) complexes. Color code: red, oxygen; light blue, nitrogen; magenta, cobalt; grey, carbon; pale yellow, fluorine; cyan, lanthanide. Hydrogen atoms and solvent molecules omitted for clarity.

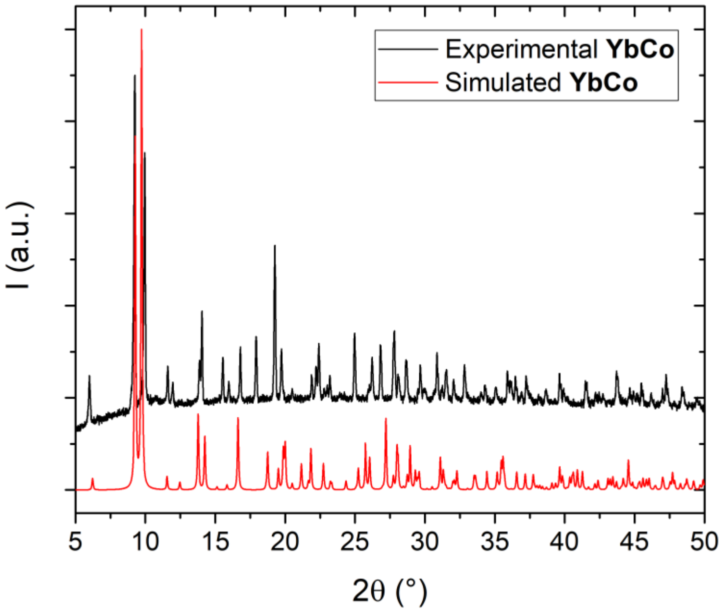

Figure S2 – Experimental (black line) and simulated (red line) powder x-ray diffractogram for the **YbCo** complex.

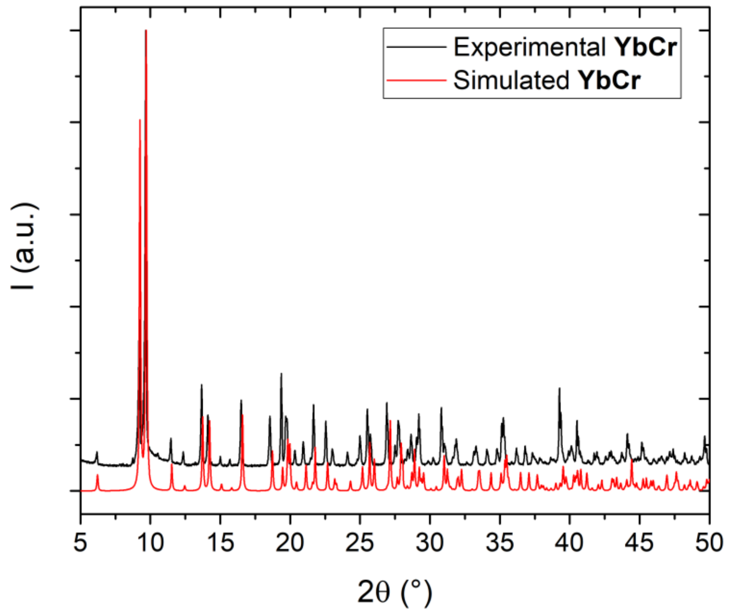

Figure S3 – Experimental (black line) and simulated (red line) powder x-ray diffractogram for the **YbCr** complex.

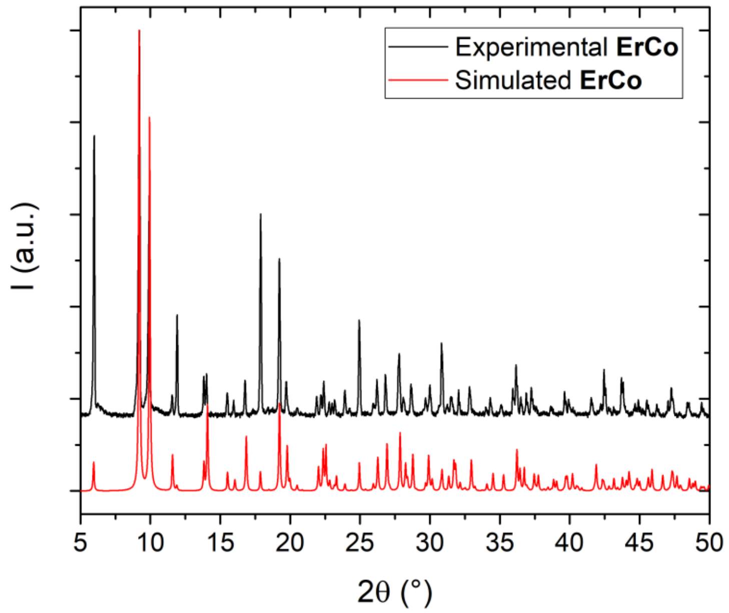

Figure S4 – Experimental (black line) and simulated (red line) powder x-ray diffractogram for the **ErCo** complex.

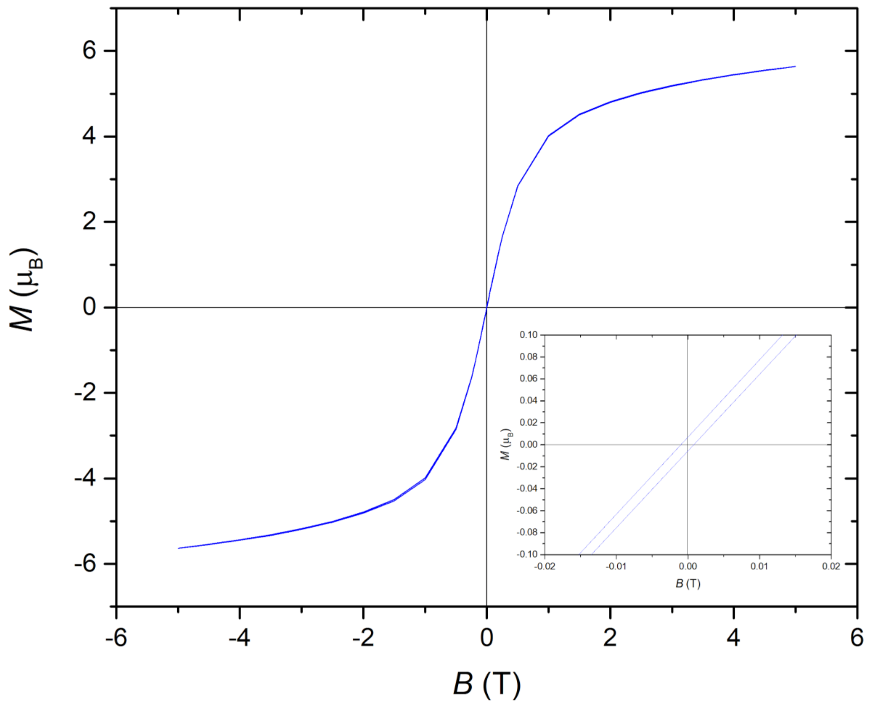

Figure S5 – Hysteresis curve acquired on polycrystalline **ErCo** at 2 K.

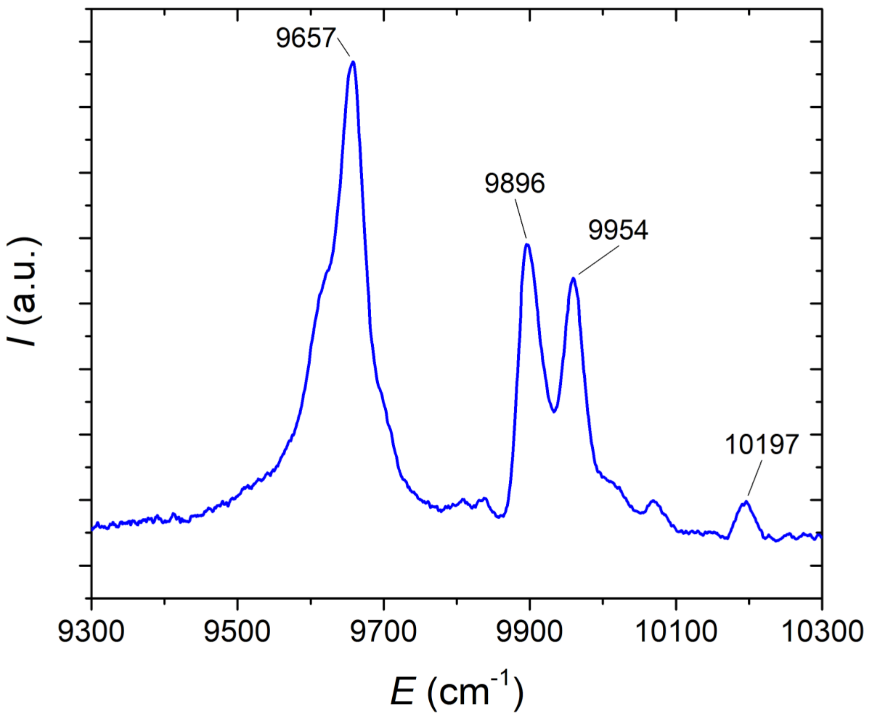

Figure S6 – Low-temperature (77 K) luminescence spectrum acquired on polycrystalline **YbCr**.

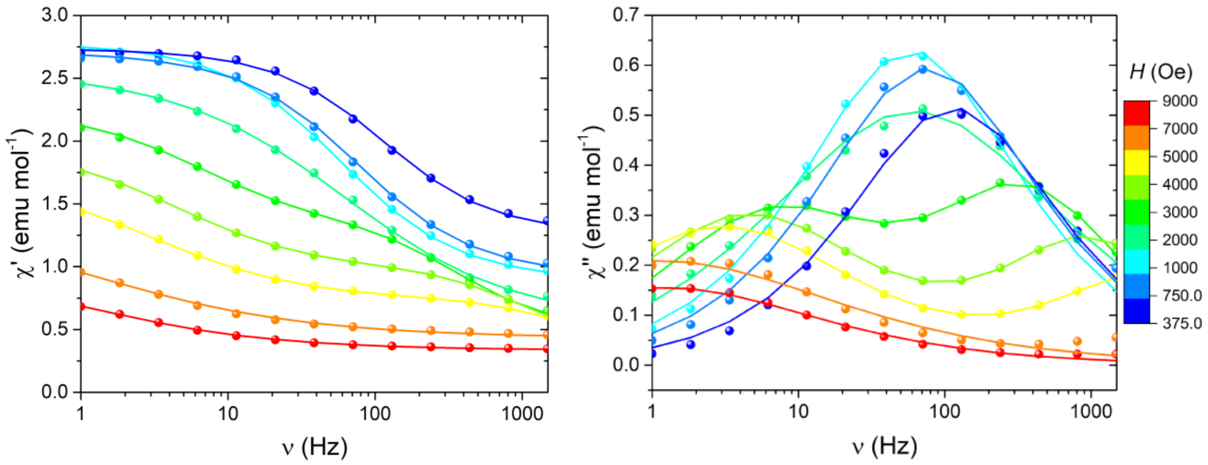

Figure S7 – Magnetic field dependence of the real (χ') and imaginary (χ'') components of the magnetic susceptibility for **ErCo** at 2K. Lines represent the best-fit curves obtained with an extended Debye model.

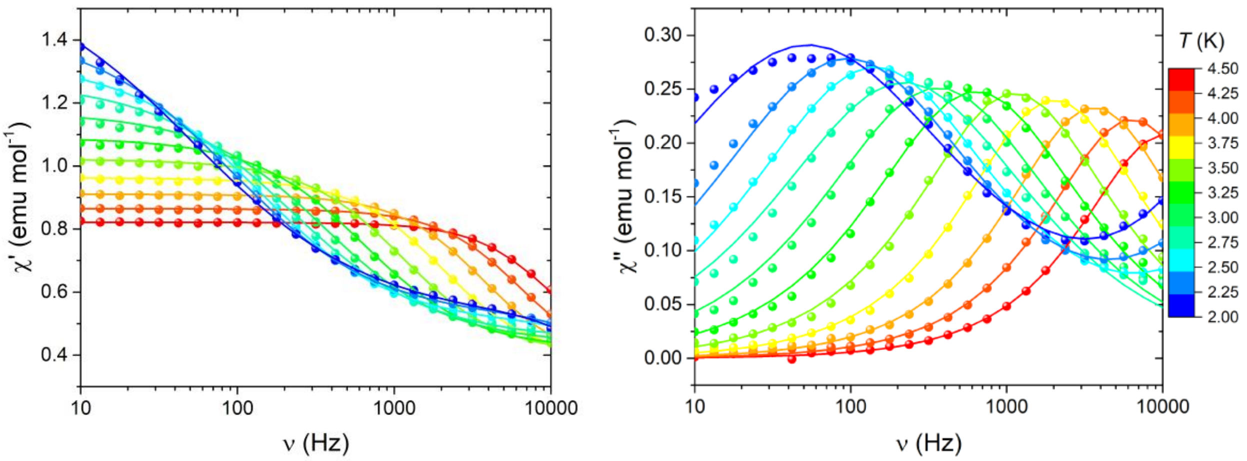

Figure S8 - Temperature dependence of the real (χ') and imaginary (χ'') components of the magnetic susceptibility for **ErCo** at 1000 Oe. Lines represent the best-fit curves obtained with an extended Debye model.

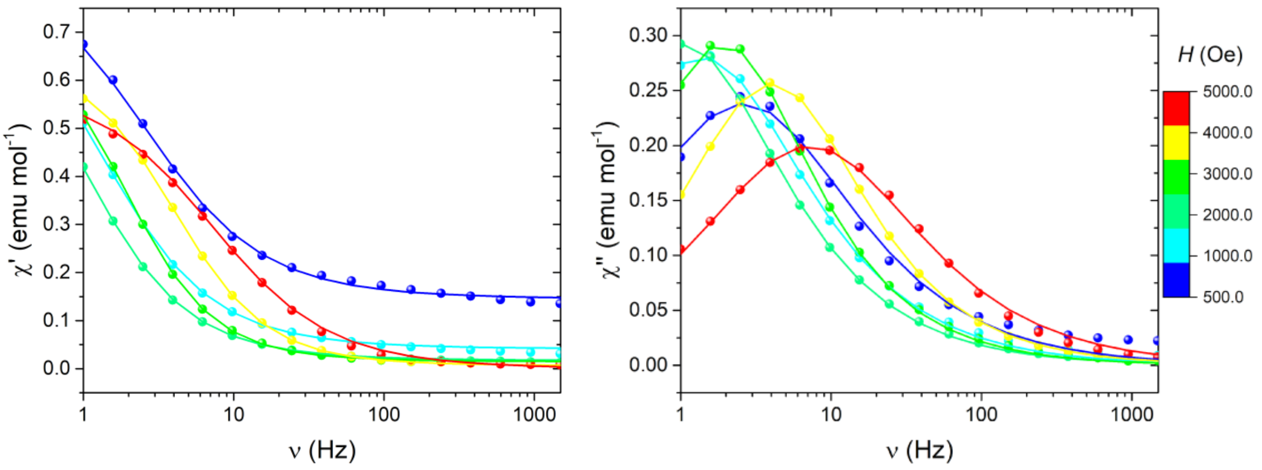

Figure S9 – Magnetic field dependence of the real (χ') and imaginary (χ'') components of the magnetic susceptibility for **YbCo** at 2K. Lines represent the best-fit curves obtained with an extended Debye model.

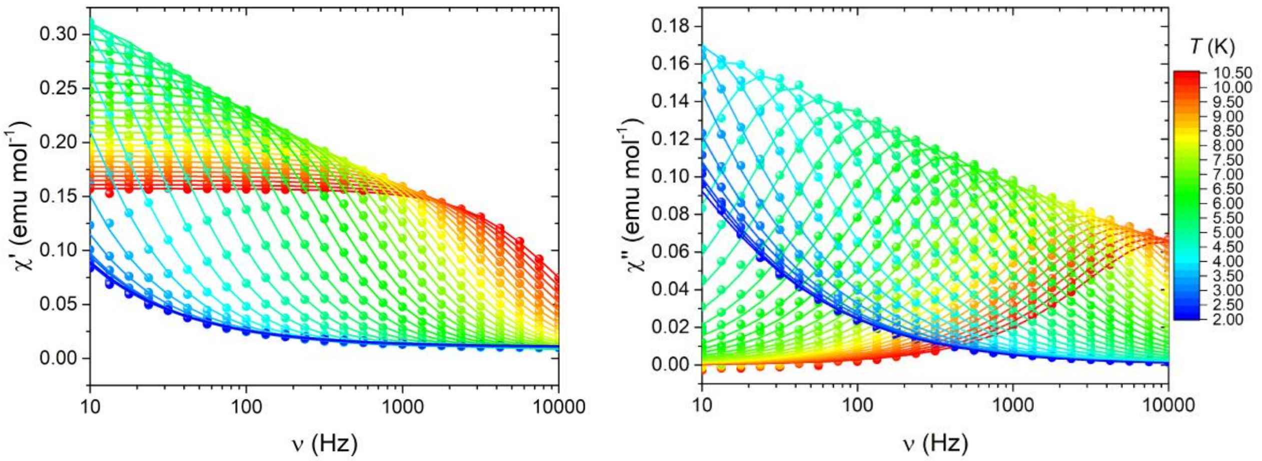

Figure S10 - Temperature dependence of the real (χ') and imaginary (χ'') components of the magnetic susceptibility for **YbCo** at 2000 Oe. Lines represent the best-fit curves obtained with an extended Debye model.

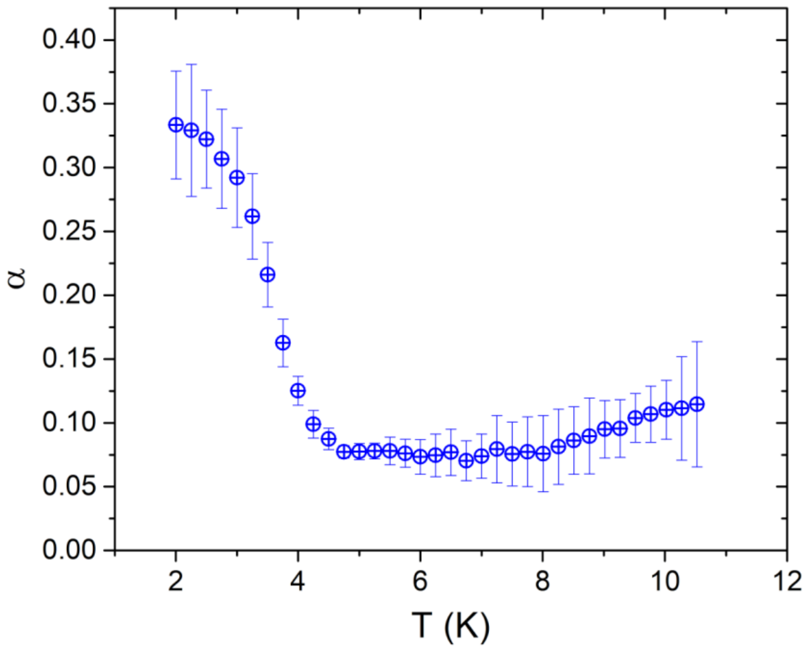

Figure S11 – Thermal dependence of the α parameter extracted from fitting the temperature dependent $\chi'(\nu)$ and $\chi''(\nu)$ curves of **YbCo** with an extended Debye model.

Supporting Tables

Table S1. Crystallographic data and refinement details **ErCo**, **YbCo** and **YbCr**.

Compound reference	ErCo	YbCo	YbCr
Chemical formula	$C_{36}H_{64}CoF_2N_8O_{18}Er$	$C_{36}H_{65}CoF_2N_8O_{19}Yb$	$C_{36}H_{65}CrF_2N_8O_{19}Yb$
Formula mass	1161.14	1183.928	1177.00
Crystal system	Tetragonal	Tetragonal	Tetragonal
a/Å	12.5635(4)	12.8534(7)	12.886(3)
b/Å	12.5635(4)	12.8534(7)	12.886
c/Å	29.7496(12)	14.1839(8)	14.194(3)
α/°	90	90	90
β/°	90	90	90
γ/°	90	90	90
Unit cell volume/Å^3	4695.7(4)	2343.3(2)	2356.9(10)
Temperature/K	100(2)	296.15	100(2)
Space group	*P*4/ncc	*P4/n*	*P4/n*
No. of formula units/unit cell, *Z*	4	2	2
Radiation type	Mo Kα	Mo Kα	Mo Kα
Absorption coefficient, μ/mm^{-1}	2.216	2.428	2.292
No. of reflections measured	5706	2804	3578
No. of independent reflections	4229	2485	3363
Data/restraints/parameters	5706/0/204	2804/0/167	3578/7/167
Final R_1 values (all data)	0.0348	0.0347	0.0300
Final $wR_2(F^2)$ values (all data)	0.0502	0.0686	0.0708
Final R_1 values ($I > 2\sigma(I)$)	0.0197	0.0276	0.0276
Final $wR_2(F^2)$ values ($I > 2\sigma(I)$)	0.0446	0.0653	0.0695
Goodness of fit on F^2	1.037	1.0347	1.101
Largest diff. peak and hole (eÅ^{-3})	1.040/-0.577	1.7694/-1.6234	2.536/-0.704
CCDC number	2441140	2441139	2441141

Table S2 – Best-fit parameters for the magnetic field and temperature dependence of relaxation frequency τ^{-1} of **ErCo**. Parameters marked with * have not been varied during the fitting procedure.

$B_1\ (s^{-1})$	$866(3)$	$\nu_1\ (cm^{-1})$	$14.0(7)$
$B_2\ (Oe^2)$	$1.41(13) \cdot 10^{-6}$	$\tau_0\ (s)$	$4.93(17) \cdot 10^{-9}$
$R_1\ (s^{-1})$	$1.03(61) \cdot 10^{6}$	$\Delta\ (cm^{-1})$	26^{*}

Table S3 – Best-fit parameters for the magnetic field and temperature dependence of relaxation frequency τ^{-1} of **YbCo**.

$B_1\ (s^{-1})$	$24.82(79)$	$R_1\ (s^{-1})$	$9.89(17) \cdot 10^{5}$
$B_2\ (Oe^2)$	$2.07(26) \cdot 10^{-6}$	$\nu_1\ (cm^{-1})$	$26.4(3)$
$D\ (Oe^{-m}\ K^{-1})$	$1.61(59) \cdot 10^{-9}$	$R_2\ (s^{-1})$	$2.25(31) \cdot 10^{8}$
m	$2.74(4)$	$\nu_2\ (cm^{-1})$	$65.2(5)$

Table S4 – Best-fit B_k^q parameters in Wybourne's notation obtained through a fitting procedure of DC magnetometry data. Parameters for **DyCo** were taken from the literature[1]. Parameters are expressed in cm^{-1}.

B_k^q	**DyCo**	**ErCo**	**YbCo**
B_2^0	-1285	-1067	-662
B_4^0	-646	-129	-167
B_6^0	-304	-61	-564
B_4^4	551	1574	446
B_6^4	-884	-574	-1006

Table S5 – Simulated energies and compositions of the $J = 15/2$ ground state for the two Er(III)DOTA archetypes. CF parameters for **ErDOTA** were taken from literature[2]. Only m_J states with a percentage higher than 5% were reported.

	**ErCo**		**ErDOTA**	
**Level**	**Energy (cm^{-1})**	**Composition (%)**	**Energy (cm^{-1})**	**Composition (%)**
$\|1>$	0	56.6 $\|+\frac{15}{2}>$<br>25.8 $\|+\frac{7}{2}>$<br>14.4 $\|-\frac{1}{2}>$	0	76.6 $\|+\frac{13}{2}>$<br>15.1 $\|+\frac{5}{2}>$<br>5.7 $\|-\frac{3}{2}>$
$\|3>$	26	11.3 $\|+\frac{13}{2}>$<br>48.5 $\|+\frac{5}{2}>$<br>40 $\|-\frac{3}{2}>$	80	13 $\|+\frac{9}{2}>$<br>54.6 $\|+\frac{1}{2}>$<br>15.2 $\|-\frac{7}{2}>$<br>17.2 $\|-\frac{15}{2}>$
$\|5>$	88	38.8 $\|+\frac{15}{2}>$<br>13.5 $\|+\frac{7}{2}>$<br>29.1 $\|-\frac{1}{2}>$<br>18.5 $\|-\frac{9}{2}>$	87	11.2 $\|+\frac{13}{2}>$<br>36.9 $\|-\frac{3}{2}>$<br>51.1 $\|-\frac{11}{2}>$
$\|7>$	149	87.1 $\|+\frac{13}{2}>$<br>9.2 $\|-\frac{3}{2}>$	149	7.4 $\|+\frac{9}{2}>$<br>16.8 $\|+\frac{1}{2}>$<br>74.6 $\|-\frac{15}{2}>$

$\|9>$	185	98.9 $\|+\frac{11}{2}>$	309	7.3 $\|+\frac{13}{2}>$<br>31.3 $\|+\frac{5}{2}>$<br>30 $\|-\frac{3}{2}>$<br>37.3 $\|-\frac{11}{2}>$
$\|11>$	227	70.2 $\|+\frac{9}{2}>$<br>24.9 $\|\pm\frac{7}{2}>$	376	30.7 $\|+\frac{7}{2}>$<br>60.3 $\|-\frac{9}{2}>$
$\|13>$	479	8.0 $\|+\frac{9}{2}>$<br>55.3 $\|+\frac{1}{2}>$<br>35.7 $\|-\frac{7}{2}>$	462	8.9 $\|+\frac{11}{2}>$<br>33.4 $\|+\frac{3}{2}>$<br>52.7 $\|-\frac{5}{2}>$
$\|15>$	509	50.7 $\|+\frac{3}{2}>$<br>48.2 $\|-\frac{5}{2}>$	485	19.3 $\|+\frac{9}{2}>$<br>24.3 $\|+\frac{1}{2}>$<br>52.8 $\|-\frac{7}{2}>$

Table S6 – Simulated energies and compositions of the $J = 7/2$ ground state for the two Yb(III)DOTA archetypes. CF parameters for **YbDOTA** were taken from literature[2]. Only m_J states with a percentage higher than 5% were reported.

	**YbCo**		**YbDOTA**	
**Level**	**Energy (cm^{-1})**	**Composition (%)**	**Energy (cm^{-1})**	**Composition (%)**
$\|1>$	0	89.1 $\|+\frac{7}{2}>$<br>10.9 $\|-\frac{1}{2}>$	0	84.1 $\|+\frac{5}{2}>$<br>15.9 $\|-\frac{3}{2}>$
$\|3>$	243	98.1 $\|+\frac{5}{2}>$	305	54.4 $\|+\frac{7}{2}>$<br>45.6 $\|+\frac{1}{2}>$
$\|5>$	301	98.1 $\|+\frac{3}{2}>$	684	45.6 $\|+\frac{7}{2}>$<br>54.4 $\|-\frac{1}{2}>$
$\|7>$	540	89.1 $\|+\frac{1}{2}>$<br>10.9 $\|-\frac{7}{2}>$	711	15.9 $\|+\frac{5}{2}>$<br>84.1 $\|-\frac{3}{2}>$

Table S7. Transition probabilities calculated based on the determined CF parameters for **ErCo** (Table S4).

	$\|1>$	$\|2>$	$\|3>$	$\|4>$	$\|5>$	$\|6>$	$\|7>$	$\|8>$	$\|9>$	$\|10>$	$\|11>$	$\|12>$	$\|13>$	$\|14>$	$\|15>$	$\|16>$
$\|1>$	0	0.97	5.14	0	3.64	2.16	0.19	0	0	0.16	0.67	0.07	0.06	0.04	0.01	0
$\|2>$	0.97	0	0	5.16	2.18	3.66	0	0.19	0.16	0	0.07	0.68	0.04	0.06	0	0.01
$\|3>$	5.14	0	0	7.41	2.57	0	1.41	1.05	0.89	0.01	0	1.15	0.08	0	0.91	0.03
$\|4>$	0	5.16	7.41	0	0	2.54	1.06	1.40	0.01	0.88	1.16	0	0	0.08	0.03	0.91
$\|5>$	3.64	2.18	2.57	0	0	3.41	3.03	0	0	0.77	0.01	3.27	0.25	0.08	0.12	0
$\|6>$	2.16	3.66	0	2.54	3.41	0	0	3.03	0.77	0	3.28	0.01	0.08	0.25	0	0.12
$\|7>$	0.19	0	1.41	1.06	3.03	0	0	0.49	3.63	0.31	0	0.20	0.05	0	0.50	0.12
$\|8>$	0	0.19	1.04	1.0	0	3.03	0.49	0	0.33	3.64	0.20	0	0	0.05	0.12	0.50
$\|9>$	0	0.16	0.89	0.01	0	0.77	3.63	0.33	0	0.09	5.09	0	0	0.47	0.07	0.04
$\|10>$	0.16	0	0.01	0.88	0.77	0	0.30	3.64	0.09	0	0	5.09	0.47	0	0.04	0.07
$\|11>$	0.67	0.07	0	1.16	0.01	3.28	0	0.20	5.09	0	0	5.43	1.62	1.75	0	0.61
$\|12>$	0.07	0.68	1.15	0	3.27	0.01	0.20	0	0	5.09	5.43	0	1.75	1.62	0.62	0
$\|13>$	0.06	0.04	0.08	0	0.25	0.08	0.05	0	0	0.47	1.62	1.75	0	7.65	9.03	0
$\|14>$	0.04	0.06	0	0.08	0.08	0.25	0	0.05	0.47	0	1.75	1.62	7.65	0	0	9.03
$\|15>$	0.01	0	0.91	0.03	0.12	0	0.50	0.13	0.07	0.04	0	0.62	9.03	0	0	9.70
$\|16>$	0	0.01	0.03	0.91	0	0.12	0.12	0.50	0.04	0.08	0.61	0	0	9.03	9.70	0

Table S8. Transition probabilities calculated based on the determined CF parameters for **ErDOTA**.[2]

	$\|1>$	$\|2>$	$\|3>$	$\|4>$	$\|5>$	$\|6>$	$\|7>$	$\|8>$	$\|9>$	$\|10>$	$\|11>$	$\|12>$	$\|13>$	$\|14>$	$\|15>$	$\|16>$
$\|1>$	0	1.42	3.08	0	4.19	2.35	0.79	0	0.05	0.13	0	0.04	0.12	0.11	0.16	0
$\|2>$	1.42	0	0	3.08	2.40	4.20	0	0.80	0.13	0.05	0.04	0	0.11	0.12	0	0.16
$\|3>$	3.08	0	0	6.62	0	3.96	3.96	1.38	0	1.18	0.04	0.53	0.16	0	0.01	0
$\|4>$	0	3.08	6.62	0	3.97	0	1.42	3.91	1.19	0	0.53	0.04	0	0.16	0	0.0
$\|5>$	4.19	2.40	0	3.97	0	0.49	0	3.00	2.38	1.53	0.56	0	0.46	0	0	0.01
$\|6>$	2.35	4.20	3.96	0	0.49	0	3.03	0	1.53	2.38	0	0.56	0	0.46	0.02	0
$\|7>$	0.79	0	3.96	1.42	0	3.03	0	0.15	0	0.17	0.89	1.46	0.25	0	0.08	0.06
$\|8>$	0	0.80	1.38	3.91	3.00	0	0.15	0	0.17	0	1.45	0.89	0	0.25	0.06	0.09
$\|9>$	0.05	0.13	0	1.19	2.38	1.53	0	0.17	0	5.98	6.61	0	0.05	2.68	0	0.02
$\|10>$	0.13	0.05	1.18	0	1.53	2.38	0.17	0	5.98	0	0	6.61	2.69	0.04	0.02	0
$\|11>$	0	0.04	0.04	0.53	0.56	0	0.89	1.45	6.61	0	0	5.28	0	0.10	1.54	3.63
$\|12>$	0.04	0	0.53	0.04	0	0.56	1.46	0.89	0	6.61	5.28	0	0.10	0	3.65	1.53
$\|13>$	0.12	0.11	0.16	0	0.46	0	0.26	0	0.05	2.69	0	0.10	0	8.64	8.54	0
$\|14>$	0.11	0.12	0	0.16	0	0.46	0	0.25	2.68	0.04	0.10	0	8.64	0	0	8.54
$\|15>$	0.16	0	0.01	0	0	0.02	0.08	0.06	0	0.02	1.54	3.65	8.54	0	0	6.76
$\|16>$	0	0.16	0	0.01	0.01	0	0.06	0.09	0.02	0	3.63	1.53	0	8.54	6.76	0

Table S9 Transition probabilities calculated based on the determined CF parameters for **YbCo** (Table S4).

	$\|1>$	$\|2>$	$\|3>$	$\|4>$	$\|5>$	$\|6>$	$\|7>$	$\|8>$
$\|1>$	0	0.03	1.17	0	0	0.14	0.26	0.52
$\|2>$	0.03	0	0	1.17	0.14	0	0.52	0.26
$\|3>$	1.17	0	0	0.15	1.85	0.10	0	0.02
$\|4>$	0	1.17	0.15	0	0.10	1.85	0.02	0
$\|5>$	0	0.14	1.85	0.10	0	0.15	2.33	0
$\|6>$	0.14	0	0.10	1.85	0.15	0	0	2.33
$\|7>$	0.26	0.52	0	0.02	2.33	0	0	2.11
$\|8>$	0.52	0.26	0.02	0	0	2.33	2.11	0

Table S10. Transition probabilities calculated based on the determined CF parameters for **YbDOTA**.[2]

	$\|1>$	$\|2>$	$\|3>$	$\|4>$	$\|5>$	$\|6>$	$\|7>$	$\|8>$
$\|1>$	0	1.07	1.04	0	0.34	0	0.93	0.71
$\|2>$	1.07	0	0	1.04	0	0.34	0.71	0.93
$\|3>$	1.04	0	0	0.55	1.32	0.66	0	0.73
$\|4>$	0	1.04	0.55	0	0.66	1.32	0.73	0
$\|5>$	0.34	0	1.32	0.66	0	0.79	0	1.56
$\|6>$	0	0.34	0.66	1.32	0.79	0	1.56	0
$\|7>$	0.93	0.71	0	0.73	0	1.56	0	1.07
$\|8>$	0.71	0.93	0.73	0	1.56	0	1.07	0

Table S11 – Comparison between the best-fit parameters for the magnetic field and temperature dependences of relaxation frequency τ^{-1} of **ErCo** and **ErDOTA**. The third column refers to the fit performed on **ErCo** data fixing the pre-exponential factors of the two Raman processes to the values previously determined for **ErDOTA**. Parameters marked with * have not been varied during the fitting procedure.

	**ErCo**	**ErDOTA**	**ErCo**
$B_1\ (s^{-1})$	$866(3)$	/	866^*
$B_2\ (Oe^2)$	$1.41(13)\cdot 10^{-6}$	/	$1.41\cdot 10^{-6^*}$
$R_1\ (s^{-1})$	$1.03(61)\cdot 10^{6}$	$1.57(4)\cdot 10^{5}$	$1.57\cdot 10^{5^*}$
$\nu_1\ (cm^{-1})$	$14.0(7)$	$13(1)$	$13.6(8)$
$\tau_0\ (s)$	$4.93(17)\cdot 10^{-9}$	/	$4.93\cdot 10^{-9^*}$
$\Delta\ (cm^{-1})$	26^*	/	26^*
$D\ (Oe^{-m}\ K^{-1})$	/	$1.35(1)$	/
m	/	$1.0(1)$	/

Table S12 – Comparison between the best-fit parameters for the magnetic field and temperature dependences of relaxation frequency τ^{-1} of **YbCo** and **YbDOTA**. The third column refers to the fit performed on **YbCo** data fixing the pre-exponential factors of the two Raman processes to the values previously determined for **YbDOTA**. Parameters marked with * have not been varied during the fitting procedure.

	**YbCo**	**YbDOTA**	**YbCo**
$B_1\ (s^{-1})$	$24.82(79)$	$394(31)$	24.82^*
$B_2\ (Oe^2)$	$2.07(26) \cdot 10^{-6}$	$2.4(5) \cdot 10^{-6}$	$2.07 \cdot 10^{-6^*}$
$D\ (Oe^{-m}\ K^{-1})$	$1.61(59) \cdot 10^{-9}$	$0.153(3)$	$1.61 \cdot 10^{-9^*}$
m	$2.74(4)$	$1.0(1)$	2.74^*
$R_1\ (s^{-1})$	$9.89(17) \cdot 10^5$	$3.57(73) \cdot 10^5$	$3.57 \cdot 10^{5^*}$
$\nu_1\ (cm^{-1})$	$26.4(3)$	$9.5(5)$	$24.5(7)$
$R_2\ (s^{-1})$	$2.25(31) \cdot 10^8$	$2.37(137) \cdot 10^7$	$2.37 \cdot 10^{7^*}$
$\nu_2\ (cm^{-1})$	$65.2(5)$	$38(3)$	$46.1(12)$

References

(1) Tacconi, L.; Manvell, A. S.; Briganti, M.; Czernia, D.; Weihe, H.; Konieczny, P.; Bendix, J.; Perfetti, M. Exploiting High Order Magnetic Anisotropy for Advanced Magnetocaloric Refrigerants. *Angew. Chemie Int. Ed.* **2025**, *137* (5), e202417582. https://doi.org/10.1002/anie.202417582.

(2) Manvell, A. S.; Pfleger, R.; Bonde, N. A.; Briganti, M.; Mattei, C. A.; Nannestad, T. B.; Weihe, H.; Powell, A. K.; Ollivier, J.; Bendix, J.; Perfetti, M. LnDOTA Puppeteering: Removing the Water Molecule and Imposing Tetragonal Symmetry. *Chem. Sci.* **2024**, *15* (1), 113–123. https://doi.org/10.1039/D3SC03928E.
